# 

**Published:** 1890-06

**Authors:** 


					﻿jmSTE, 1880.
OLD SERIES
VoL XXV
NEW SERrtSB
Vol. VII.-Ito.Aj
Z^®L,S^
& 1855 0

lEDIGAl^lURWb
pwURNAK



W. F. WESTMORELAND, M.D.	<
H. V. M. MILLER, M.D., LL.D.
VIRGIL 0. HARDON, M.D.	1
F. W. McRAE, M.D., M’n’g’ng Ed.
Published Hr james rharrison&ce
VC	. a. "Atlanta .ga. jfc
				

